# Tetrachloromethane-Degrading Bacterial Enrichment Cultures and Isolates from a Contaminated Aquifer

**DOI:** 10.3390/microorganisms3030327

**Published:** 2015-07-02

**Authors:** Christian Penny, Christelle Gruffaz, Thierry Nadalig, Henry-Michel Cauchie, Stéphane Vuilleumier, Françoise Bringel

**Affiliations:** 1 CNRS Génétique Moléculaire, Génomique, Microbiologie, Université de Strasbourg, UMR 7156 UNISTRA-CNRS, 28 rue Goethe, 67083 Strasbourg, France; E-Mails: christian.penny@list.lu (C.P.); c.gruffaz@unistra.fr (C.G.); nadalig@unistra.fr (T.N.); vuilleumier@unistra.fr (S.V.); 2 Environmental Research and Innovation (ERIN), Luxembourg Institute of Science and Technology (LIST), 5 avenue des Hauts-Fourneaux, 4362 Esch/Alzette, Luxembourg; E-Mail: henry-michel.cauchie@list.lu

**Keywords:** C1 compound biodegradation, tetrachloromethane, carbon tetrachloride, bacterial communities, bacterial enrichment cultures, co-metabolism, chlorinated solvents, *Pelosinus* sp. TM1

## Abstract

**Abstract**: The prokaryotic community of a groundwater aquifer exposed to high concentrations of tetrachloromethane (CCl_4_) for more than three decades was followed by terminal restriction fragment length polymorphism (T-RFLP) during pump-and-treat remediation at the contamination source. Bacterial enrichments and isolates were obtained under selective anoxic conditions, and degraded 10 mg·L^−1^ CCl_4_, with less than 10% transient formation of chloroform. Dichloromethane and chloromethane were not detected. Several tetrachloromethane-degrading strains were isolated from these enrichments, including bacteria from the *Klebsiella* and *Clostridium* genera closely related to previously described CCl_4_ degrading bacteria, and strain TM1, assigned to the genus *Pelosinus*, for which this property was not yet described. *Pelosinus* sp. TM1, an oxygen-tolerant, Gram-positive bacterium with strictly anaerobic metabolism, excreted a thermostable metabolite into the culture medium that allowed extracellular CCl_4_ transformation. As estimated by T-RFLP, phylotypes of CCl_4_-degrading enrichment cultures represented less than 7%, and archaeal and *Pelosinus* strains less than 0.5% of the total prokaryotic groundwater community.

## 1. Introduction

Tetrachloromethane (carbon tetrachloride, CCl_4_) has been widely used for decades as industrial degreasing agent, pesticide, flame retardant and in dry cleaning [1]. Prolonged large-scale use of CCl_4_ has caused significant environmental soil and groundwater contamination. Because of its hazardous effects on human health [2] and its implication in depletion of atmospheric ozone, industrial production and use of CCl_4_ is regulated under the Montreal Protocol since 1987. Nevertheless, worldwide CCl_4_ emissions remain high (up to 39 Gg/year between 2000 and 2012 [3,4]). CCl_4_ is highly recalcitrant and shows the longest half-life for abiotic degradation among halogenated aliphatic compounds [5]. Microbial CCl_4_ degradation occurs exclusively under anoxic conditions [6], and can be mediated, often extracellularly, by many biomolecules including corrinoid [7,8], quinone [9,10] and humic acid [11] derivatives; cytochromes [12]; nickel- and zinc-containing porphinoids [13]; riboflavin [14]; and siderophores [15]. Biodegradation of CCl_4_ by taxonomically diverse, both strict and facultative anaerobic bacteria, as well as by methanogenic Archaea, was reported under a wide range of conditions including denitrification, sulfate reduction, iron reduction, fermentation, acetogenesis and methanogenesis [6,16]. CCl_4_-degrading microorganisms growing with CCl_4_ as the sole carbon source have not been reported, but several studies showed some incorporation of CCl_4_ carbon as biomass via CCl_4_ degradation intermediates or products [6,17,18]. Nevertheless, CCl_4_ transformation is highly toxic to degrading organisms, notably because of the formation of reactive free radicals upon dehalogenation, which lead to formation of stable deleterious adducts both in the cytoplasm and in the cell membrane [6]. Processes that would allow productive CCl_4_ metabolism without such toxic effects would be expected to confer a selective advantage to their host in CCl_4_-contaminated environments. Therefore, isolating new microorganisms able to degrade CCl_4_, and stimulating the development of such organisms, or adding selected microbial strains known for their sustained CCl_4_-degrading activity in contaminated environments, represent attractive goals for cost-effective *in situ* remediation of CCl_4_-contaminated sites [6,19].

The purpose of this study was to investigate the CCl_4_ biodegradation potential of the prokaryotic community from a sub-surface aquifer located in Alsace, France, which was heavily contaminated by an accidental spill of 4000 liters of CCl_4_ in 1970. Infiltration into the groundwater, which remained undetected for over 20 years, led to the gradual development of a 12-km long plume. Maximum CCl_4_ concentrations at the contamination source exceeded more than thousand-fold the WHO drinking water guideline value of 4 μg·L^−1^. Physico-chemical “pump-and-treat” remediation at the contamination source was performed between August 2006 and June 2009, at which point operational costs became critical. Here, terminal restriction fragment length polymorphism (T-RFLP) [20] was used to identify changes in diversity in the microbial community over three sampling campaigns carried out at the pollution source over a 15-month period during pump-and-treat remediation, and to follow several CCl_4_-degrading enrichment cultures obtained from samples taken at the contamination source. This led to the isolation of the novel CCl_4_-degrading bacterial strain *Pelosinus* sp. TM1.

## 2. Materials and Methods

### 2.1. Chemicals

Unless otherwise stated, all chemicals and reagents were of analytical grade and obtained from Sigma-Aldrich Co. (Saint-Louis, MO, USA) or Thermo Fisher Scientific (Waltham, MA, USA).

### 2.2. Sampling of Groundwater and Concentration of Microorganisms

Groundwater from the studied CCl_4_-polluted site was collected in October 2007 (T1), April 2008 (T2) and December 2008 (T3), *i.e.*, 14, 20 and 28 months after the start of “pump-and-treat”. Sampling was performed at the location of the initial spill by diverting 100 (T1 and T2) or 1000 (T3) liters of pumped groundwater before physico-chemical remediation. pH and redox potentials were measured using a pH 330i pH-meter (WTW, Weilheim, Germany) equipped with a SenTix Mic or a SenTix ORP electrode for pH or E_h_ determination, respectively. Mineral composition was determined by groundwater analyses carried out by the Centre d’Analyses et de Recherches in Strasbourg. Samples were concentrated by tangential filtration using a Prep/Scale TFF cartridge with a molecular weight cut-off of 30 kDa (samples T1 and T2; Millipore, Billerica, MA, USA), or with a Sartocon slice ultrafiltration set equipped with 5 polyethersulfone membranes of 0.1 m^2^ (sample T3; cut-off 30 kDa; Sartorius, Göttingen, Germany). Sample volumes were further reduced from 300–400 mL to 10–20 mL by Centricon Plus-70 centrifugal filter devices (T1 and T2; Millipore), or by centrifugation at 10,000 g for 30 min in an Optima L90K ultracentrifuge equipped with a fixed angle 45Ti rotor (T3; Beckman, Brea, CA, USA). Cell densities were estimated using a DM 4000B epifluorescence microscope (Leica, Wetzlar, Germany) and fluorescent (SYBR Green I) intracellular DNA staining as described [21].

### 2.3. Cultivation Methods

Three culture media with different carbon and energy sources and electron acceptors were used. MBM medium (containing (per L) 2 g yeast extract, 2 g casitone and 10 mL methanol) corresponds to *Methanosarcina barkeri* medium n°120a from the German culture collection DSMZ. C medium contains 10 g yeast extract and 0.5 g tryptone per L and was prepared as described [22]. *Pseudomonas stutzeri* KC [23] and *Methanosarcina barkeri* DSM 1538 [24] were used as positive controls for microbial degradation of CCl_4_. Simulated groundwater medium (SGW), modified from [25] to reduce the concentration of chloride, contained (per L of deionized water) 0.35 g Na_2_SiO_3_·5H_2_O, 0.16 g Na_2_CO_3_, 0.006 g Na_2_SO_4_, 0.02 g KOH, 0.15 g MgSO_4_·7H_2_O, 0.0125 g Ca(NO_3_)_2_·4H_2_O, 1.6 g Na_2_HPO_4_, 0.8 g KH_2_PO_4_ and 1 mL of trace elements solution. pH was adjusted to 7.5–7.8 with 10 M NaOH prior to autoclaving at 121 °C for 10 min. Individual salt solutions and trace elements were added separately as sterile 100× concentrated stock solutions after autoclaving. When used as solid medium, 15 g·L^−1^ bacteriological agar (Euromedex, Souffelweyersheim, France) was added. After sterilization, medium for anoxic growth was transferred into 500 mL glass infusion bottles (Glasgerätebau Ochs, Bovenden, Germany) sealed with Teflon-coated rubber stoppers (Apodan, København, Denmark), then flushed under a continuous flow of nitrogen (Linde Gas, Saint-Priest, France) for 1 h. After transfer to an anoxic glovebox (Jacomex, Saint-Priest, France), media were reduced by addition of 0.3 g·L^−1^ Na_2_S·9H_2_O and 0.3 g·L^−1^ l-cysteine·HCl. The redox indicator resazurin (in aqueous 1 mM solution; Riedel-de Haën, Germany) was added to liquid media at a final concentration of 1 μM.

Liquid enrichment cultures (5 mL) were carried out at 25 °C in 17 mL Hungate tubes sealed with Viton rubber stoppers (Glasgerätebau Ochs, Bovenden, Germany) in MBM, C and SGW media. Several CCl_4_ concentrations (0, 10 and 200 mg·L^−1^), carbon sources (methanol, acetate, lactate, succinate, glucose, yeast extract, casitone or tryptone), and electron acceptors (oxygen, nitrate, sulfate, bicarbonate or organic compounds) were tested (Table S1). Oxic incubations were performed in a Microtron rotary shaker at 100 rpm (Infors, Bottmingen, Switzerland). Cultures under anoxic conditions were prepared in an anoxic glove box in Hungate tubes that were degassed using a vacuum pump connected to a nitrogen flow circuit (H. Lüdi + Co., Regensdorf, Switzerland) before addition of O_2_-depleted medium. Carbon and energy sources and electron acceptors were added as 100× concentrated sterile stock solutions in water as summarized in Table S1. Saturated aqueous CCl_4_ solutions (800 mg·L^−1^) were prepared using ultrapure CCl_4_ (purity > 99.9%; Fluka) and added last to culture media. Inoculation of enrichment cultures was done with 100 μL aliquots of concentrated groundwater samples from campaigns T1 and T2, representing between 0.5 and 1.0 L of the initial groundwater samples. Growth was measured at 600 nm directly in Hungate culture tubes with a Libra S6 spectrophotometer equipped with a 16 mm culture tube adapter (Biochrom, UK). Enrichment cultures for selected samples were obtained under the same conditions by successive reinoculation with 100 μL of stationary phase cultures (OD 0.2–1.0).

### 2.4. Gas Chromatography

CCl_4_ was quantified using a CP 3800 gas chromatograph connected to a flame ionization detector (GC-FID; Varian, Palo Alto, CA, USA). Headspace aliquots (500 μL) were collected from sealed Hungate culture tubes with a gastight 1750 syringe (Hamilton, Franklin, MA, USA) and injected onto the GC column (CP-Sil 5 CB, length 15 m; Varian). Separation of volatile compounds was achieved by isothermal elution at 30 °C for 1 min, followed by a linear temperature gradient (20 °C min^−1^) up to 220 °C. Injector and detector were maintained at 220 °C (splitless mode) and 300 °C, respectively, with nitrogen (N_2_) as the make-up gas (Linde Gas). Peak areas were analyzed with Galaxie Workstation software (Varian) and expressed as percentage of CCl_4_ compared to an abiotic reference tube prepared under the same conditions.

### 2.5. Determination of CCl_4_ Degradation Rates and Chloride Production

Specific CCl_4_ degradation rates in growing cultures of dechlorinating enrichments and of strain TM1 were calculated by determining the amount of CCl_4_ degraded per day and per mg of total cellular protein upon culturing under the respective conditions described in Table S1. Average degradation rates were determined per day of culture, and normalized per unit of cellular protein (average of at least three measurements during growth by the method described below). Similarly, rates of CCl_4_ degradation and chloride production were determined in cell suspensions of strain TM1 grown in SGW medium. Cell cultures (50 mL OD_600_ 0.15–0.20) were centrifuged at 10,000 *g* for 15 min, resuspended (equivalent to 0.9 mg total protein) in 5 mL 10× diluted SGW medium in 30 mL sealed anoxic flasks under a nitrogen atmosphere, and exposed to 65 μM (10 mg·L^−1^) CCl_4_ at 25 °C. Headspace samples were taken for GC measurements as described above, and 550 μL aliquots of the cell suspension were removed for determination of protein content and chloride concentration. Briefly, aliquots were spun down and an appropriate dilution of the supernatant analyzed by ion chromatography on an AS18-AG18 column (Dionex, Sunnyvale, CA, USA) eluted with 34 mM NaOH at 1 mL per minute. Protein measurements were performed on cell pellets resuspended in 0.1 M NaOH, lysed at 95 °C for 10 min and centrifuged at 12,000 rpm for 5 min. Aliquots of the resulting solutions were analyzed with the bicinchoninic acid kit (Sigma) following the manufacturer’s protocol, using bovine serum albumin (BSA) as a standard. For analysis of CCl_4_ degradation in spent medium, culture supernatants were separated from cells of a 500 mL culture grown in SGW medium by centrifugation for 15 min at 7650 *g* in a Sorvall Evolution RC centrifuge equipped with a SS-34 rotor (Thermo Fisher Scientific). Supernatants were filter-sterilized using 0.22 μm cellulose acetate syringe filters (VWR, Fontenay-sous-Bois, France). Corresponding cell pellets were washed and resuspended in an equivalent volume of 50 mM phosphate buffer, pH 7.5. Heat treatment was performed by autoclaving (121 °C, 10 min), and oxygen-exposed samples were obtained by contact with air for 90 min under sterile conditions. Part of the oxygen-exposed supernatant fraction was cysteine/Na_2_S-treated by addition of 0.3 g·L^−1^ Na_2_S·9H_2_O and 0.3 g·L^−1^ l-cysteine·HCl. Degradation rates in supernatants were expressed as μg CCl_4_ day^−1^ per mL of bacterial culture, normalized for a culture at OD_600_ = 1.0.

### 2.6. Bacterial Phenotyping

Substrate utilization profiling of strain TM1 was performed under anaerobic conditions with an AN MicroPlate (Biolog, Hayward, CA, USA) according to the manufacturer’s recommendations, except that incubation was set to 25 °C for 72 h in an anoxic glove box. Staining of spores was carried out as described [26], except that a fuchsine solution (basic fuchsine 10 g·L^−1^, phenol 50 g·L^−1^, ethanol 100 mL·L^−1^) was used for staining instead of 1% aqueous safranin.

### 2.7. DNA Purification

Total DNA from environmental samples or enrichment cultures was purified and isolated from 10^7^ to 10^9^ cells trapped in agarose plugs [20]. DNA concentrations were estimated with a NanoDrop ND-1000 (Thermo Fisher Scientific). DNA from single colonies on solid media was extracted from approximately 10^8^ cells suspended in 40 μL sterile ultrapure water or NaOH 0.05 M, and heated at 95 °C for 10 min. The resulting solution was cooled on ice for 5 min, diluted (1/15) in sterile ultrapure water and used as template for PCR amplification.

### 2.8. T-RFLP Analysis

PCR reaction mixtures, cycles and primers for bacterial T-RFLP were previously described [20]. Primers 6-FAM-21f and 958r [27] were used for T-RFLP analysis of the archaeal 16S rRNA gene. Preparation of PCR-amplified 0.9 kb 16S rRNA gene fragments and T-RFLP analysis were previously described [20]. The T-RF database was constructed using the “Microbial community analysis III” interface (MiCA III; [28]), with the following parameters: primers 27f and 926r, restriction enzymes *Alu*I and *Hha*I, RDP release 10 update 27 (700,829 good quality bacterial SSU 16S rRNA fragments), and 10 mismatches allowed within 15 bases from 5′ end of primers. Data obtained with different fluorochromes for internal marker and sample fragments permitted determination of the total number of T-RFs and their relative proportion. The peak integration threshold was set to 500 ppm of the total integrated fluorescence. Bacterial diversity was evaluated using the Shannon diversity index (*H′*) [29], as follows:

(1) $$H\prime =-\sum_{i=1}^{S}p_{i}\cdot \ln(p_{i})$$

where *p_i_* is the relative area of T-RFLP peak *i* and *S* is the total number of integrated peaks (richness) in the sample. The distribution of T-RFLP peak areas was also evaluated using a measure of evenness (*E*):

(2) $$E=\frac{H\prime }{\ln(S)}$$

The maximum value *E* = 1.0 would describe a sample with equal frequencies of all peaks in its T-RFLP profile.

### 2.9. Denaturing High Performance Liquid Chromatography (D-HPLC) Analysis

D-HPLC was performed as described [20], using a gradient of 58% to 67% buffer B in 18 min, at a temperature of 59.5 °C and a flow rate of 0.45 mL·min^−1^, for separation of 16S rRNA gene fragments.

### 2.10. Fluorescence in Situ Hybridization

Groundwater samples were fixed with 2% formaldehyde solution for 2 h. After washing with phosphate buffer saline solution (PBS), samples were stored in a 1:1 PBS/ethanol solution at −20 °C until used. For hybridization, 20 μL were filtered through 0.22 μm polycarbonate filters (GE Healthcare Europe, Vélizy-Villacoublay, France). Archaea were detected with the specific Cy3 monolabeled “S-D-Arch-0915-a-A-20” probe [30]. After staining with 4,6-diamidino-2-phenylindole (DAPI), total cells were counted with appropriate fluorescent filters using a DM 4000B epifluorescence microscope (Leica, Wetzlar, Germany).

### 2.11. 16S rRNA Gene Cloning, Sequencing and Analysis

PCR reactions from enrichment cultures were performed with primers 27f and rd1 (5′-AAGCTTAAGGAGGTGATCCAG-3′), adapted from [31]. Zero Blunt TOPO PCR cloning kit (Invitrogen) was used for cloning obtained 16S rRNA gene PCR amplicons. Selection of individual clones for sequencing was based on *Alu*I or *Hha*I restriction profile polymorphism of PCR amplicons obtained from cloned fragments with T3 and T7 universal primers, and visualized on NuSieve 3:1 Agarose gels (2% equivalent agarose; Tebu-bio, Le Perray-en-Yvelines, France). Sequences of PCR products, purified with ExoSAP-IT (USB Corporation, USA) following the manufacturer’s protocol, were obtained with primers 27f, 534r or rd1 on an ABI Prism 3130 XL sequencer (Applied Biosystems, Waltham, MA, USA). Similarity searches of the 16S rRNA gene sequences were performed using Blast and the Ribosomal Database Project [32].

### 2.12. Accession Numbers

The 16S ribosomal RNA gene sequences obtained for selected bacterial strains enriched from the studied site were submitted to the EMBL database under accession numbers FN689722 to FN689728 and KP219716.

## 3. Results

### 3.1. CCl_4_-Degrading Enrichments and Isolation of *Pelosinus sp*. TM1

**Figure 1:**
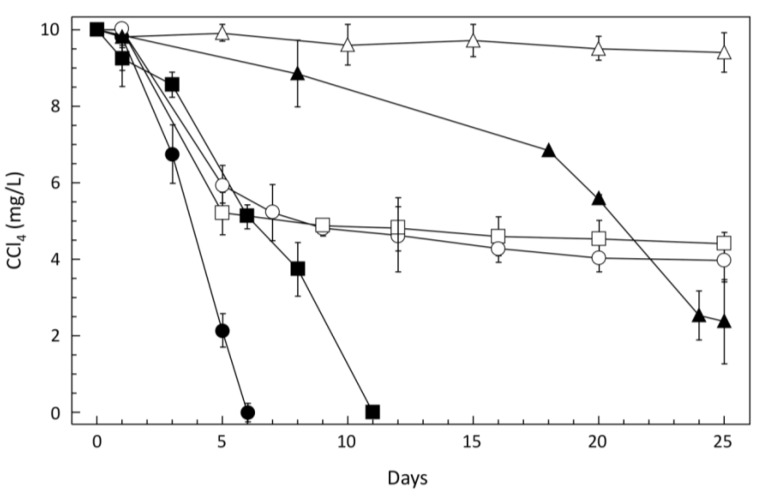
CCl_4_ degradation by enrichment cultures from the contaminated aquifer. Enrichments C1 (filled circles) and C2 (filled squares) were grown in C medium, and enrichments C3 (filled triangles); C4 (open squares); C5 (open circles) and a non-dechlorinating control consortium (open triangles) in MBM medium (see Materials and Methods and Tables S1–S2 for details). Error bars represent the standard error of 3 technical replicates.

To identify CCl_4_-degraders among the prokaryotic community of concentrated groundwater samples, enrichment cultures under 33 different selective conditions were set up for each of the collected samples in campaigns T1 and T2 (11 different conditions, in the presence of 0, 10 and 200 mg·L^−1^ CCl_4_; Table S1). Removal of CCl_4_ in enrichment cultures was only observed anaerobically in nutrient-rich C and MBM media with initial CCl_4_ concentrations of 10 mg·L^−1^, and growth was observed 24 to 72 h after inoculation. Delayed and slower growth without degradation activity was observed at 200 mg·L^−1^. In five enrichment cultures (designated C1 to C5), degradation of CCl_4_ was complete between six and 11 days after inoculation. Sustained CCl_4_ degradation in successive cultures was assessed by inoculating aliquots of stationary phase CCl_4_-degrading enrichments into fresh medium. Two types of degradation curves were observed after successive transfers (Figure 1). Initially, cultures C4 and C5 degraded CCl_4_ fastest in the first enrichment cultures, but eventually lost their capacity for CCl_4_ degradation upon successive inoculations under the same culture conditions (Table S2; Figure 1). Evaluation of the diversity of Bacteria and Archaea in the five CCl_4_-degrading enrichment cultures by 16S rRNA gene T-RFLP analysis evidenced: (i) different fingerprints for each enrichment with exclusively bacterial content (no Archaea); (ii) shifts in community structure after successive transfers; and (iii) predominant phylotypes identified by database comparisons ([28], Table 1).

**Table 1:** 16S rRNA-based taxonomic affiliation of bacterial strains in CCl_4_-degrading enrichments.

Name	Proposed affiliation/Accession Number ^a^	Best Hit ^a^	
		Taxon/Accession Number	Identity (%)/Length (bp)
Cultivated isolates			
Strain TM1	*Pelosinus* sp./FN689722,FN68972, KP219716	*Pelosinus propionicus* TmPM3/AM258974	99/1549
Strain TM2	*Klebsiella sp.*/FN689724	*Klebsiella oxytoca* M1/CP008841	98/1437
Strain TM5	*Peptostreptococcaceae bacterium*/FN689725	*Clostridium ruminantium* Neferana 2/KJ722512	99/1382
Strain C5_1	*Staphylococcus sp.*	*Staphylococcus aureus* sub sp. *Aureus*/BA000017	99/535
Uncultivated OTUs			
C1_2, C2_3, C3_3	No sequence data	No T-RFLP database match	n.a.^b^
C2_1	No sequence data	Uncultured actinobacteria/AJ575500, AJ575552, AJ575544, AJ575549, AJ575559; Uncultured gamma-proteobacterium/AB294927	n.a. n.a.
C3_2	Clostridiales/FN689728	Uncultured Clostridiaceae bacterium B-LO-T0_OTU18/FM204961	99/439
C4_1	Bacteroidetes/FN689727	Uncultured bacterium ASSO-106/JN391680	97/402
C4_2	Lactobacillales/FN689726	Uncultured *Trichococcus sp*. D4R-61/AB331457	100/479

^a^ NCBI Blast and RDP database [32]. When no sequence data were available, T-RFLP database analysis was performed using a size confidence interval of ±1 nt [28]; ^b^ n.a., not applicable.

Strains TM1, TM2 and TM5 were isolated as single colonies from corresponding CCl_4_-degrading enrichments plated out on solid C medium (Table S2). Strain TM2 was affiliated to *Klebsiella* sp. basing on its 16S rRNA gene sequence (Table 1). Several enterobacteria catalyzing CCl_4_ degradation were described previously, including *Klebsiella pneumoniae* strain L17 (98% sequence identity with strain TM2), a dissimilatory iron-reducing bacterium (DIRB) capable of CCl_4_ degradation under anoxic conditions in presence of ferric oxides and humic substances [33]. Strain TM5 showed a 16S RNA gene sequence similar to representatives of *Peptostreptococcaceae* (Table 1), which comprise *Acetobacterium woodii*, *Moorella thermoacetica* and *Clostridium* sp. strain TCAIIB, previously described to dechlorinate CCl_4_ by a transformation process involving corrinoids [18,22,34]. Initially, strain TM5 displayed the fastest degradation rate observed in this study (Table S2), but its ability to degrade CCl_4_ was progressively lost upon transfer of the enrichment culture. Strain TM1, the most stable CCl_4_-degrading strain isolated in this work, was affiliated to the genus *Pelosinus* basing on 16S rRNA gene sequence analysis (Table 1).

Strains TM1, TM2 and TM5 and representatives of the other taxa identified in dechlorinating consortia represented less than 7% of the prokaryotic groundwater community analyzed by T-RFLP, with proportions ranging from 0.1% to 2.0% of the groundwater community (Table S2; Figure S1A). Diversity indices of the prokaryotic population of the CCl_4_-contaminated aquifer from water samples of the contamination source obtained at months 14, 21 and 29 after beginning of pump-and-treat showed little difference, confirming that major shifts in endogenous bacterial populations did not occur during “pump-and-treat” (Figure S1; Table S3).

### 3.2. Characteristics of CCl_4_-Degrading *Pelosinus* sp. TM1

**Figure 2:**
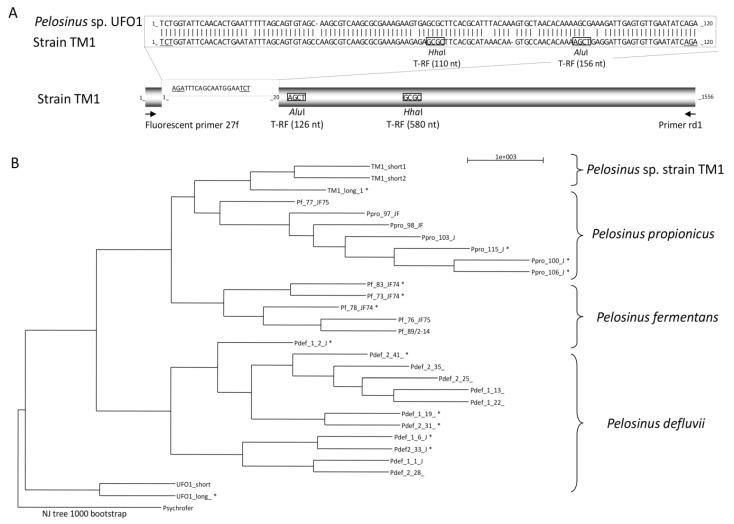
Taxonomic affiliation and 16S rRNA gene heterogeneity of strain TM1. (**A**) Schematic representation of the sequence variation in the three identified copies of the 16S rRNA gene of strain TM1, and of 5′-end AluI and HhaI fragments detected in T-RFLP analysis. (**B**) Phylogenetic analysis of the multiple 16S rRNA sequences of isolated strains of the genus *Pelosinus* [35] including the three sequences from strain TM1, with the longer sequence indicated by a star.

Strain TM1 was shown to contain at least three different ribosomal RNA operons (Table 1). Although a single colony type was observed on solid medium, T-RFLP profiles with two AluI T-RF bands of 126 and 156 nt and two HhaI T-RFs of 110 and 580 nt were repeatedly observed (Table S2). Complementary D-HPLC [20] and cloning steps allowed the identification of two 16S rRNA sequences of 1556 bp differing by only five bases, as well as a longer sequence of 1656 bp*.* The three 16S rRNA genes shared 99% overall sequence identity except at the 5′-end of the gene, in which the longer sequence featured a 120 bp sequence instead of the 20 bp sequence shared by the two shorter sequences. Additional HhaI and AluI restriction sites in the longer sequence insert (Figure 2A) provided the explanation for the observed T-RFLP fingerprints. Taxonomically closest strains from the genus *Pelosinus* also feature sequence inserts at the 5′-end of their 16S rRNA genes (Figure 2B) [35,36].

**Figure 3:**
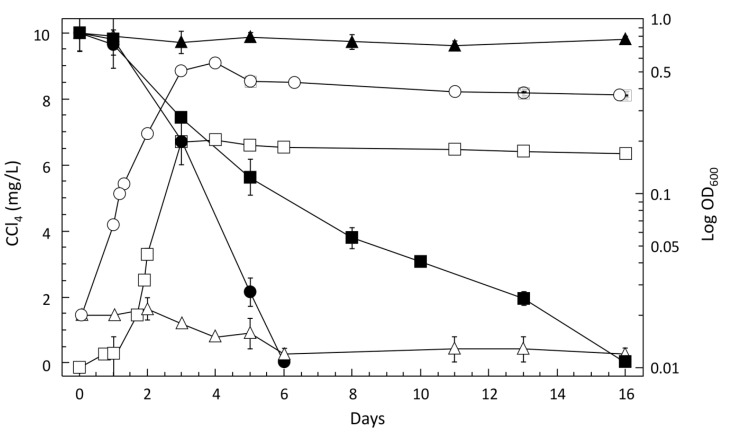
CCl_4_ degradation during growth of *Pelosinus* sp*.* TM1. Optical density (open symbols) and CCl_4_ degradation (filled symbols); cultivation with 10 mg·L^−1^ CCl_4_ in anoxic C medium (circles), C medium exposed to oxygen for one week (triangles), and anoxic SGW medium (squares). Error bars represent the standard error of 3 technical replicates.

*Pelosinus* sp. TM1 is a strictly anaerobic bacterium, with Gram-positive rod-shaped cells 3–7 μm in length and 0.4–0.6 μm in diameter. It grew under anoxic conditions with a generation time of about 12 h in the three media used in this study (Table S1, Figure 3). No growth was observed after heat treatment (100 °C for 8 min), and spores were not detected. In C medium at 25 °C, strain TM1 reached an OD_600 nm_ of about 0.6 in stationary phase (Figure 3), and retained its viability under anoxic conditions after exposure to oxygen for one week (incubation under sterile conditions in an air-exposed rotary shaker at 100 rpm at 25 °C). Strain TM1 metabolized 79 carbon sources out of the 95 of the Biolog “anaerobe identification test panel”. Carbon sources that were not utilized included *N*-acetyl-d-glucosamine, *N*-acetyl-mannosamine, adonitol, d-arabitol, i-erythritol, d-glycerol, d-trehalose, glyoxylic acid, itaconic acid, urocanic acid, l-alanyl-l-threonine, l-valine, l-aspartic acid, inosine, thymidine and uridine. The range of growth-supporting carbon sources of strain TM1 was identical to that of the corresponding type strain *P. propionicus* TmPM3^T^ [37], except that strain TM1 was additionally able to metabolize succinate, propionate, acetate and formate, but unable to use d-glycerol.

CCl_4_ degradation by *Pelosinus* sp. TM1 paralleled growth in C medium, with a degradation rate of 2.2 μg CCl_4_ day^−1^ mg protein^−1^ (Figure 3; Table 2). A similar specific degradation rate of 2.3 ± 0.3 μg CCl_4_ day^−1^ mg protein^−1^ was found after growth under more oligotrophic groundwater-simulating conditions (SGW medium supplemented for growth yield purposes with pyruvate (10 mM) as the potential electron donor, 10 times diluted yeast extract compared to C medium, and nitrate (10 mM) as nitrogen source). CCl_4_ degrading ability of strain TM1 was further assessed by estimating the amounts of chloride production from CCl_4_ degradation in cell suspensions of cultures grown in SGW medium with 10 mM pyruvate. Rates of CCl_4_ removal (0.8 nmol ± 0.2 CCl_4_ h^−1^ mg^−1^) and chloride production (2.8 nmol ± 0.4 Cl^−^ h^−1^ mg^−1^) were compatible with a stoichiometry of four chloride ions produced per CCl_4_ consumed (Figure S2). Notably, *Pelosinus* sp. TM1 was unable to degrade other chlorinated methanes (Figure S3).

**Table 2:** Degradation of CCl_4_ by *Pelosinus* sp. TM1.

Culture Fraction ^a^		Degradation Rate	
		μg mL (OD 1.0) ^−1^ day^−1^	μg mg protein^−1^ day^−1^
Liquid culture in C medium		1.9 ± 0.6	2.2 ± 0.7
Resuspended cells	Phosphate buffer ^b^	9.1 ± 1.1	10.6 ± 1.3
	Phosphate buffer ^b^, heat-treated	13.6 ± 0.9	15.8 ± 1.0
	Phosphate buffer ^b^, heat-treated, oxygen-exposed	<0.1	<0.1
Supernatant	Filter-sterilized	1.4 ± 0.3	n.d. ^c^
	Filter-sterilized, heat-treated	1.6 ± 0.4	n.d.
	Filter-sterilized, oxygen-exposed	<0.1	n.d.
	Filter-sterilized, oxygen-exposed, cysteine/Na_2_S-treated	1.4 ± 0.4	n.d.

^a^ Total initial CCl_4_: 50 μg in 5 mL liquid volume (10 mg·L^−1^); ^b^ 50 mM; ^c^ n.d., not determined.

Living cells of *Pelosinus* sp. TM1 were not required for CCl_4_ degradation, since filter-sterilized supernatants of stationary phase cultures displayed a similar CCl_4_ degradation rate as cell suspensions of the strain (Table 2). The ability of cultures and cell suspensions of *Pelosinus* sp. TM1, as well as of cell-free supernatants, to degrade CCl_4_ following heat treatment, oxygen exposure or addition of the reducing agents l-cysteine·HCl or Na_2_S was tested. Exposure to oxygen suppressed CCl_4_ degradation (cell-free supernatant and resuspended cells in phosphate buffer; Table 2). Treatment of oxygen-exposed supernatants with reducing agents restored CCl_4_ degradation to levels similar to those of non-treated anoxic supernatants. Heat treatment of supernatants had no effect on CCl_4_ degradation activity. Taken together, these data indicate that *Pelosinus* sp. TM1 synthesized a thermostable molecule that can be released from cells in the supernatant, is redox-active (*i.e.*, oxidized by oxygen and reduced by sulfide) and was at least in part responsible for CCl_4_ degradation.

## 4. Discussion

To our knowledge, *Pelosinus* sp. TM1 is the first isolate of the genus shown to be capable of dehalogenation. Nevertheless, *P. fermentans* R7 was shown to provide an essential corrinoid cofactor for *Dehalococcoides* reductive dehalogenases in a trichloroethene-dechlorinating syntrophic consortium [38], and several strains of the genus *Pelosinus* were isolated from sites contaminated with halogenated compounds including chlorinated alkanes [35,38,39,40]. *Pelosinus* sp. TM1 afforded stable and complete dehalogenation of CCl_4_ (Table 2, Table S2), at rates comparable to those previously observed for both *Escherichia coli* K12 and for a consortium of various anaerobic bacteria under anoxic conditions (0.2 and 0.3 μg day^−1^ mg protein^−1^, respectively) [41,42].

### CCl_4_ Degradation by *Pelosinus* sp. TM1 is a Co-Metabolic Process

A CCl_4_-degrading microbial process in which CCl_4_ is used as a carbon or energy source for growth has not been identified [6,41,42]. Several lines of evidence suggest that *Pelosinus* sp. TM1 also involves a co-metabolic process. In terms of carbon, the quantities and rate of degraded CCl_4_ under the tested conditions will at most only make a minor contribution to the biomass produced (e.g., 3 mg protein in the presence of 3.9 μg CCl_4_ carbon). In terms of energy, and although growth-promoting metabolism of CCl_4_ may be thermodynamically favorable [6], the obtained data suggest that it is unlikely that CCl_4_ is used by *Pelosinus* sp. TM1 as an electron acceptor in energy-generating metabolism for growth. First, comparing the molar ratio of electron donor pyruvate (10 mM) to the amounts of CCl_4_ added to cultures in SGW medium (65 μM), CCl_4_ would have been growth limiting when used as an electron acceptor. Second, considerable amounts (>60%) of the initial CCl_4_ remained once *Pelosinus* sp. TM1 reached stationary phase under the chosen culture conditions. Finally, CCl_4_ degradation also occurred in spent medium, where it resisted heat treatment (Table 2), further suggesting that, at least in part, dehalogenation of CCl_4_ in cultures of *Pelosinus* sp. TM1 may be an abiotic process. Several examples of extracellular degradation of CCl_4_ mediated by microbially produced low molecular weight compounds are known [6]. For example, under denitrifying conditions, *P. stutzeri* KC excretes the transition metal-chelator pyridine-2,6-bis(thiocarboxylate) (PDTC) which, when complexed to Cu(II), mediates extracellular dechlorination of CCl_4_ [15]. In addition, vitamin B_12_ and related corrinoid cofactors, which are produced and released to the extracellular compartment by a variety of microorganisms, also catalyze degradation of CCl_4_ [7,34,43,44]. The heat-stable molecule produced by *Pelosinus* sp. TM1 mediating CCl_4_ degradation might indeed be related to corrinoid analogues, and such cofactors were shown to be produced by *Pelosinus* strains [38], but further investigations will be required to determine its identity.

## 5. Conclusions

Diversity analysis of the prokaryotic community of an aquifer contaminated by CCl_4_ for decades and enrichment and isolation of CCl_4_-degrading bacteria in the presence of CCl_4_ showed that only a minor fraction of the bacteria from this environment were capable of CCl_4_ degradation in the laboratory. Among those, strains from genera *Klebsiella* and *Clostridium* had already been associated previously with CCl_4_ degradation. In contrast, *Pelosinus* sp. TM1 isolated here is the first representative of the *Pelosinus* genus shown to sustain CCl_4_ degradation.

## Supplementary Files

Supplementary File 1
