# Supplementary material for: Tetrachloromethane-Degrading Bacterial Enrichment Cultures and Isolates from a Contaminated Aquifer
Source: Microorganisms. 2015 Jul 2;3(3):327–43. doi: 10.3390/microorganisms3030327 (PMC5023247; doi:10.3390/microorganisms3030327)
Supplement: Supplementary File 1 [file microorganisms-03-00327-s001.doc]

Supplemental Information


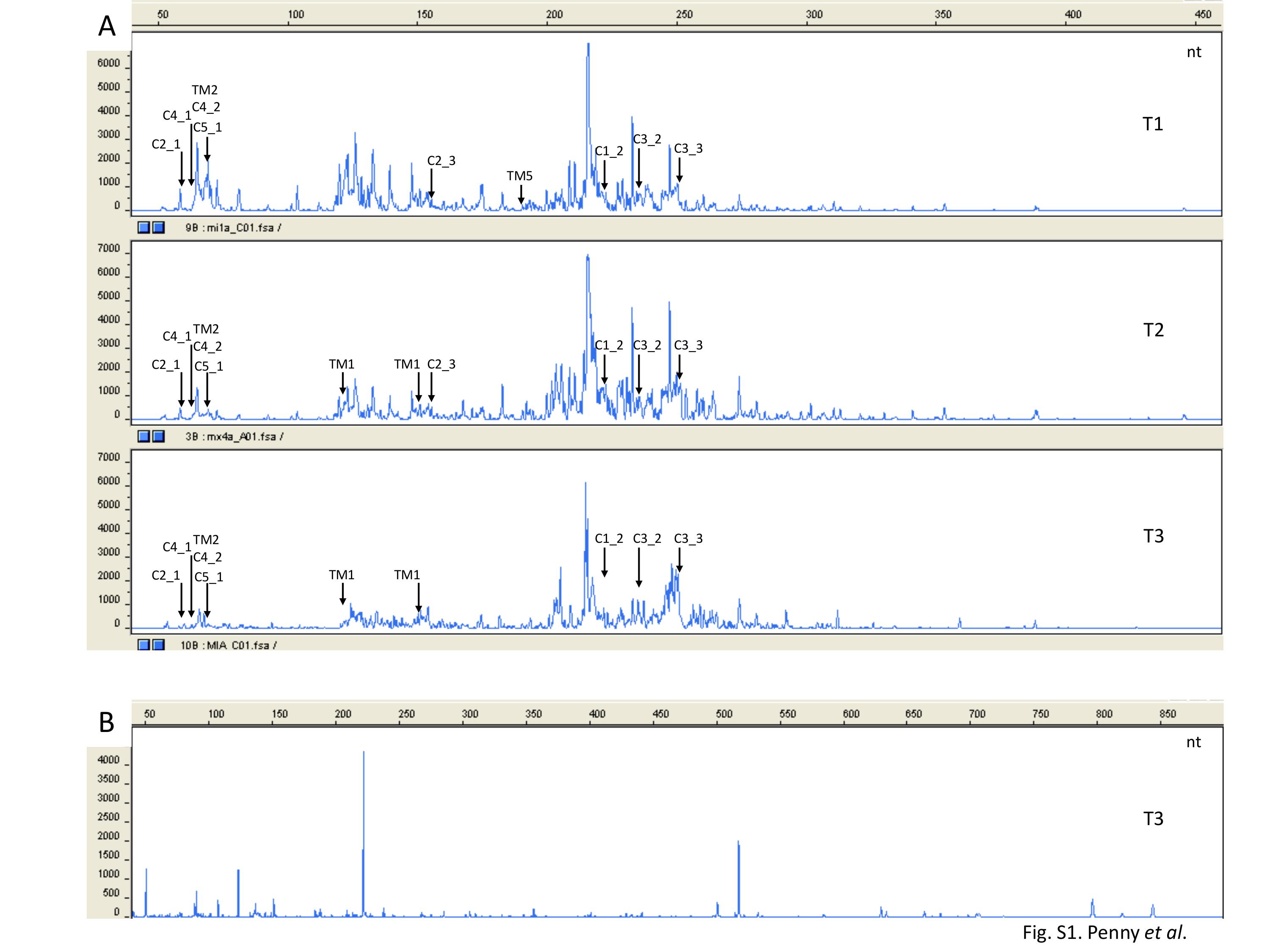


**Figure S1. T-RFLP genotyping of prokaryotic communities.** Bacterial (**A**) and archaeal (**B**) *Alu*I restriction enzyme profiles of amplified 16S rRNA gene fragments for the
3 groundwater sampling campaigns T1, T2 and T3, performed 14, 21 and 28 months, respectively, after begin of physico-chemical remediation at the pollution source. Labels indicate the expected positions of OTUs subsequently detected in CCl_4_-degrading enrichments and strains (Table1). Total area of peaks in each of the 3 chromatograms of part A is the same.


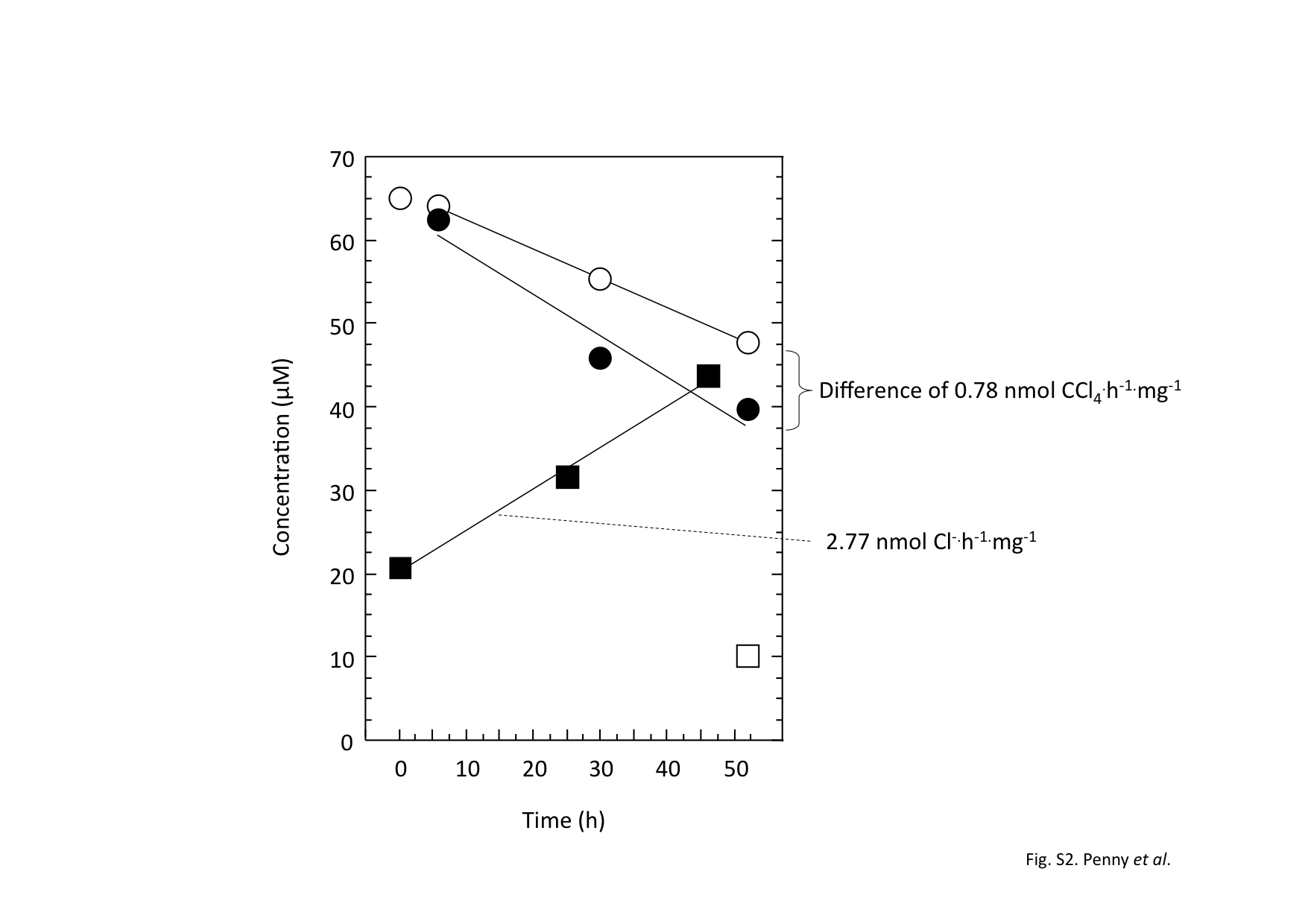


**Figure S2. Mass balance for CCl_4_ dehalogenation by *Pelosinus* sp. TM1**. A cell suspension of strain TM1 (equivalent to 0.9 mg total protein) grown in SGW medium with 10 mM pyryvate was incubated at 25 °C under nitrogen in 5 mL 10× diluted SGW medium (full symbols). CCl_4_ (circles) and chloride concentration (squares) was measured following addition of 65 μM (10 mg/L) CCl_4_. In the control without cells, CCl_4_ decreased in an identical parallel incubation experiment (empty symbols). Rates of CCl_4_ degradation and chloride production were calculated from the slopes (CCl_4_ abiotic: 0.355 μM/h; CCl_4_ with strain TM1: 0.497 μM/h; chloride production: 0.498 μM/h) as indicated. Results from a representative experiment are shown.


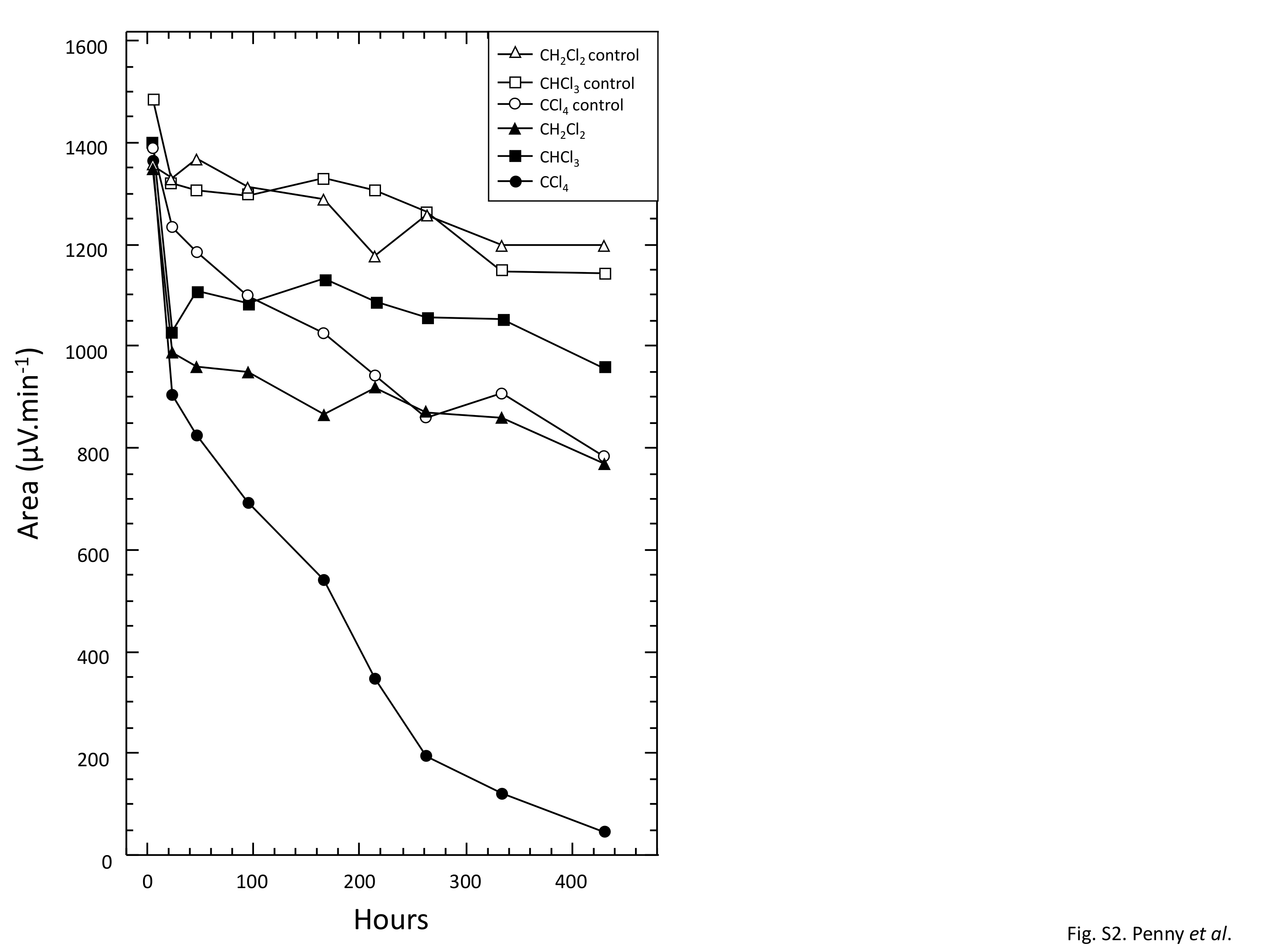


**Figure S3. Dehalogenation activity of *Pelosinus* sp. TM1 with chlorinated methanes**. Chlorinated methanes CCl_4_ (circles), CHCl_3_ (squares) and CH_2_Cl_2_ (triangles) were investigated. An aliquot of stationary phase strain TM1 (0.8 mL OD_600_ = 0.15–0.2 after growth in SGW medium with 10 mM pyruvate and 0.5 g·L^−1^ yeast extract) was added to 40 mL fresh medium and aliquoted to eight 16mL Hungate tubes kept under a nitrogen atmosphere (5 mL cell suspension to each tube). Chlorinated methanes were then added at an initial concentration of 65 μM to each tube (filled symbols), and to identical Hungate tubes containing 5 mL medium under nitrogen to which no strain TM1 culture was added (open symbols). All tubes were incubated at 25 °C.

**Table S1. Conditions used for enrichment of CCl_4_-degrading strains**

| **Carbon Source and Electron Donor** | **Medium** | **Electron Acceptor** | **Targeted Metabolism** | **CCl4-Degrading Culture ^a^** |
| --- | --- | --- | --- | --- |
| Acetate (20 mM) | SGW | Oxygen | Aerobic respiration | None |
| Yeast extract (10 g·L^−1^),  tryptone (5 g·L^−1^) | C | Oxygen  Nitrate (20 mM) | Aerobic respiration Denitrification | None |
| Acetate (20 mM) | SGW | Oxygen Nitrate (20 mM) | Aerobic respiration Denitrification | None |
| Lactate (30 mM) | SGW | Nitrate (20 mM)  Sulfate (20 mM) | Denitrification  Sulfate reduction | None |
| Acetate, methanol, succinate, glucose (10mM each) | SGW | Nitrate (20 mM)  Sulfate (20 mM) | Denitrification  Sulfate reduction | None |
| Acetate (30 mM) | SGW | Nitrate (20 mM)  Sulfate (20 mM) | Denitrification  Sulfate reduction | None |
| Acetate (30 mM) | SGW | Bicarbonate (10 mM) | Methanogenesis | None |
| Acetate, methanol, succinate, glucose (10mM each) | SGW | Nitrate, sulfate, bicarbonate (10 mM each) | Anaerobic respiration | None |
| Yeast extract (10 g·L^−1^),  tryptone (5 g·L^−1^) | C | Organic compounds | Anaerobiosis | C1, C2 ^b^ |
| Yeast extract (2 g·L^−1^),  casitone (2 g·L^−1^), methanol (250 mM) | MBM | Organic compounds, bicarbonate (10 mM) | Anaerobiosis Methanogenesis | C3, C4, C5 ^b^ |
| Yeast extract (2 g·L^−1^),  casitone (2 g·L^−1^), lactate (30 mM) | MBM | Organic compounds, bicarbonate (10 mM) | Anaerobiosis Methanogenesis | None |

^a^ Replicate enrichment cultures;^b^ Non-dechlorinating consortia were also obtained under the same
culture conditions.

Enrichment cultures were set up in the presence or the absence of CCl_4_ at 10 and 200 mg·L^−1^.

The neutral pH (7.1 ± 0.1) and relatively high redox potential of +200 mV (±25 mV) measured in groundwater samples prompted us to select a range of culture conditions allowing for growth of a large variety of prokaryotes ranging from aerotolerant to strict anaerobes. Final medium pH was 7.5–7.8 (SGW medium), 7.5 (C medium) and 6.5–6.8 (MBM medium), respectively. Although sulfate
(0.48 mM) and nitrate (0.23 mM) were predominant anions in the aquifer, enrichment cultures under sulfate-reducing conditions did not lead to detectable growth or CCl_4_ degradation. Similarly, growth was observed under denitrifying conditions, but no CCl_4_ degradation was detected whereas *Pseudomonas stutzeri* KC [1] grown as a control under the same conditions, displayed a CCl_4_ degradation rate of 14.5 ± 2.0 μg day^−1^ mg protein^−1^. In a further control experiment, *Methanosarcina barkeri* DSM 1538 grew under the tested methanogenic conditions in MBM and also degraded CCl_4_ (14.0 ± 8.5 μg day^−1^ mg protein^−1^) as described previously [2].

**Table S2. Characteristics of CCl_4_-degrading enrichments.**

| **Culture name** | **CCl_4_ degradation ^a^** | | | **Strain/OTU**  **Abundance ^c^** | **Groundwater Abundance (%)** | **Population Structure** | | | |
| --- | --- | --- | --- | --- | --- | --- | --- | --- | --- |
|  | **Rate**  **(μg day^−1^ mg protein^−1^)** | | **Extent ^b^ (%)**  **4th Enrich.** |  |  | **Size RFs (nt)** | | **Proportion (%)** | |
|  | **1st Enrich.** | **4th Enrich.** |  |  |  | **AluI** | **HhaI** | **1st Enrich.** | **4th Enrich.** |
| C1 | 2.6 ± 0.2 | 2.2 ± 0.7 | 100 | **TM1** | 0.4 ± 0.3 | 126, 156 | 580, 110 | 32, 20 | 65, 18 |
|  |  |  |  | C1_2 | 0.8 ± 0.2 | 226 | 585 | 15 | <7 ^d^ |
| C2 | 1.4 ± 0.1 | 1.4 ± 0.1 | 100 | C2_1 | 0.2 ± 0.1 | 67 | 567 | 15 | 14 |
|  |  |  |  | **TM2** | 0.6 ± 0.2 | 74 | 372 | 65 | 47 |
|  |  |  |  | C2_3 | 0.2 ± 0.2 | 159 | 327 | 9 | 20 |
| C3 | 7.7 ± 3.1 | 1.2 ± 0.2 | 76 ± 10 | **TM2** | 0.6 ± 0.2 | 74 | 372 | 27 | 83 |
|  |  |  |  | C3_2 | 1.2 ± 0.1 | 240 | 387 | 52 | 6 |
|  |  |  |  | C3_3 | 1.2 ± 0.3 | 255 | 374 | 6 | <0.1 |
| C4 | 7.2 ± 1.1 | 2.0 ± 0.1 | 60 ± 6 | C4_1 | 1.9 ± 0.9 | 71 | 92 | 40 | 2 |
|  |  |  |  | C4_2 | 0.9 ± 0.6 | 75 | 586 | 28 | 42 |
|  |  |  |  | C4_3 | n.d ^e^. | 145 | n.d. | <0.1 | 35 |
| C5 | 12.2 ± 2.7 | 3.2 ± 0.4 | 54 ± 5 | C5_1 | 0.6 ± 0.2 | 74 | 239 | 60 | 5 |
|  |  |  |  | **TM5** | 0.2 ± 0.1 | 192 | 881 | 34 | 70 |
|  |  |  |  | C5_3 | n.d. | 227 | n.d. | <0.1 | 15 |

^a^ 10 mg·L^−1^ initial CCl_4_ concentration in enrichment (Enrich.) cultures. Chloroform (CHCl_3_) formation from CCl_4_ did not exceed 10% of the initial CCl_4_ in any culture. If produced, dichloromethane (CH_2_Cl_2_) and chloromethane (CH_3_Cl) were below the detection limit of our GC method; ^b^ After 4 transfers and 25 days. All initial enrichment cultures had 100% CCl_4_ degradation after 6 to 11 days; ^c^ In bold, isolated dechlorinating strains; Operational taxonomic units (OTU) termed Cx_y where Cx refers to the corresponding consortium. Subpopulations of less than 5% of the total for both enrichments are not shown; ^d^ After 8 transfers; ^e^ n.d., not determined.

**Table S3.** Bacterial diversity in groundwater contaminated with tetrachloromethane.

| **Sampling Campaign** | **Cell Density ^a^**  **(10^4^ cellsmL^−1^)** | **T-RFLP Analysis ^b^** | | | |
| --- | --- | --- | --- | --- | --- |
|  |  | ***Alu*I** | | ***Hha*I** | |
|  |  | **OTU Number** | ***E* Index** | **OTU Number** | ***E* Index** |
| T1 | 6.0 ± 1.0 | 166 | 0.85 | 249 | 0.88 |
| T2 | 4.4 ± 1.6 | 173 | 0.85 | 271 | 0.90 |
| T3 | 5.4 ± 0.6 | 178 | 0.87 | 221 | 0.86 |

^a^ Cell densities in the contaminated aquifer were estimated by epifluorescence microscopy. Archaea were estimated by FISH as 0.2%–0.5% of the total prokaryotic community. *Alu*I T-RFLP analysis of the Archaea revealed 91 different T-RFs (see Figure S1B); ^b^ Each OTU corresponds to a specific T-RF peak
(see Materials and Methods). Of the 236 operational taxonomic units (OTUs) defined by restriction of amplicons with *Alu*I, 110 (representing 82% of the overall bacterial diversity in proportion) were found in all three samples. Similarly, with *Hha*I, a total of 122 OTUs out of 375 (representing 62% of the T-RF proportional diversity) were common to the three samples.

Reference

1. Lee, C.H.; Lewis, T.A.; Paszczynski, A.; Crawford, R.L. Identification of an extracellular catalyst of carbon tetrachloride dehalogenation from *Pseudomonas stutzeri* strain KC as pyridine-2,6-bis(thiocarboxylate). *Biochem. Biophys. Res. Commun.* **1999**, *261*, 562–566.

2. Novak, P.J.; Daniels, L.; Parkin, G.F. Enhanced dechlorination of carbon tetrachloride and chloroform in the presence of elemental iron and *Methanosarcina barkeri*, *Methanosarcina thermophila*, or *Methanosaeta concillii*. *Environ. Sci. Technol.* **1998**, *32*, 1438–1443.

© 2015 by the authors; licensee MDPI, Basel, Switzerland. This article is an open access article distributed under the terms and conditions of the Creative Commons Attribution license (http://creativecommons.org/licenses/by/4.0/).
